# Proton therapy for pediatric diencephalic tumors

**DOI:** 10.3389/fonc.2023.1123082

**Published:** 2023-05-05

**Authors:** Adam J. Grippin, Susan L. McGovern

**Affiliations:** Department of Radiation Oncology, University of Texas MD Anderson Cancer Center, Houston, TX, United States

**Keywords:** pediatric brain tumor, diencephalon, radiation, craniopharyngioma, low grade glioma, pituitary adenoma, germ cell tumor, proton therapy

## Abstract

Diencephalic tumors tend to be low grade tumors located near several critical structures, including the optic nerves, optic chiasm, pituitary, hypothalamus, Circle of Willis, and hippocampi. In children, damage to these structures can impact physical and cognitive development over time. Thus, the goal of radiotherapy is to maximize long term survival while minimizing late effects, including endocrine disruption leading to precocious puberty, height loss, hypogonadotropic hypogonadism, and primary amenorrhea; visual disruption including blindness; and vascular damage resulting in cerebral vasculopathy. Compared to photon therapy, proton therapy offers the potential to decrease unnecessary dose to these critical structures while maintaining adequate dose to the tumor. In this article, we review the acute and chronic toxicities associated with radiation for pediatric diencephalic tumors, focusing on the use of proton therapy to minimize treatment-related morbidity. Emerging strategies to further reduce radiation dose to critical structures will also be considered.

## Introduction

The diencephalon is a deep-seated midline region of the brain that includes the optic nerves, optic chiasm, pituitary gland, thalamus, hypothalamus, third ventricle, and Circle of Willis, and is very close to surrounding brain structures including the hippocampi (1). Many tumors arise in the diencephalon including optic pathway/hypothalamic glioma, craniopharyngioma, germ cell tumors, Langerhans cell histiocytosis, and pituitary adenomas (1). Diencephalic tumors often result in symptoms from involvement of the structures from which they arise or those that are nearby.

Treatment often includes definitive local therapy with maximal safe resection with or without radiation (2–9). However, local therapy is difficult due to the close proximity of adjacent critical structures. Fortunately, when these typically low-grade tumors arise in pediatric patients, survival outcomes are good relative to other intracranial tumors, with three year overall survival (OS) greater than 90% for all of these entities (7, 8, 10–13). However, each treatment modality is associated with significant morbidity (7, 8, 10–13). The late effects of radiation are driven by the close proximity of adjacent structures resulting in significant acute and chronic toxicity including endocrine dysfunction, loss of visual acuity, and vasculopathy. Given the excellent survival outcomes in these patients, the focus in the field has shifted toward methods to reduce long term treatment morbidity.

By allowing increased precision, proton therapy promises to reduce normal tissue toxicity without compromising treatment outcomes (14–16). The theoretical benefit of this effect is quantified by dosimetric studies evaluating the dose distribution to critical brain structures with protons and photons in a variety of intracranial malignancies. These studies have found a significant reduction in dose to the normal brain with proton therapy, with the largest decreases to the cochlea and uninvolved normal brain (i.e. temporal lobe) for the diencephalic tumors (16).

In the following sections, the available clinical data regarding the use of proton therapy in patients with diencephalic tumors will be reviewed. Each section begins with a discussion of the acute and chronic toxicities associated with the use of photon-based radiation for pediatric diencephalic tumors and then highlights the use of proton therapy to avoid or reduce these toxicities. The conclusion offers a brief discussion of next generation techniques, including those in clinical utilization and preclinical investigation.

## Optic pathway/hypothalamic glioma

Low grade gliomas can arise throughout the brain but often occur in the diencephalon. The most common to develop in the diencephalon are optic pathway glioma and pilocytic astrocytoma. Due to the difficulty of accessing tumors in this location, gross total resection (GTR) and sub-total resection (STR) are usually not feasible. Instead, patients are treated with definitive radiation. Chemotherapy is often used to delay the use of radiation until the patient reaches 10 years of age (9, 11). However, targeted therapies have an increasing role as a primary therapeutic modality (17).

While pilocytic astrocytomas and optic pathway glioma generally arise spontaneously, a significant portion of optic pathway gliomas result from NF1 mutations. Radiation is avoided in patients with NF1-associated glioma, who have significantly worse radiation toxicity including a five-fold increased incidence of occlusive vasculopathy (18). This review will therefore focus on the effects of radiation in patients with non-NF1-associated optic pathway/hypothalamic glioma.

### Photon-based radiation for optic pathway/hypothalamic glioma

Although randomized clinical trials are not available, a series of excellent prospective and retrospective analyses provide estimations of the toxicity of photon-based radiation and the benefits of proton therapy for this disease.

One illustrative review completed at the University of Florida included 29 pediatric patients with LGG (15 of which were diencephalic) who were treated with photon-based radiation (40-55Gy) between 1970 and 2004 with a median follow up of 17.8 years. In this subset of patients, tumor control is very good with 10-year local control of 74% and 10-year OS of 85%. Although radiation was initially tolerated well, 65% of the 23 survivors developed grade 3+ toxicity including 30% with significant cognitive disability. In addition, hearing loss was seen in 1 (4%), endocrine deficiency in 6 (26%), secondary tumor in 5 (22%), cerebrovascular event in 3 (13%), radio-necrosis in 1 (4%), and hydrocephalus in 2 (9%). Most strikingly, 14% of children in this study ultimately died due to treatment related complications. Importantly, the authors note that “all serious toxicity occurred >10 years after treatment”, which suggests that the timeline of follow up on these patients must be extensive to determine chronic toxicity (10). This finding is supported by a population based analysis that found that patients with pediatric diencephalic LGG tumors who are initially managed with upfront radiation and survive more than five years without recurrence have a 3-fold increase in incidence of late death compared to those who were managed without upfront radiation (19).

These data are also supported by an even larger retrospective review of 361 patients with LGG (63.5% pilocytic astrocytoma; 41% diencephalic), treated with radiation, surgery, or chemotherapy with 15 year follow-up which demonstrated similarly favorable OS (86%) and PFS (55%). The majority of these patients underwent maximal total resection, with 53% STR and 40% GTR. Local control was similar at 40%. As in the University of Florida experience, the patients whose tumors resolved with treatment unfortunately experienced significant 15-year cumulative incidence of growth hormone (GH) deficiency (60%), seizure (26.5%), blindness (27%), and CN deficit (24%). These toxicities occurred more frequently for those with progressive disease. These adverse events occurred with gradual incidence distributed almost evenly over the 15 year period except for hyperinsulinism, which occurred most frequently in years 10-15 after radiation, and GHD, which occurred most frequently in the first ten years after radiation but less frequently after this time. As expected, diencephalic tumor location was an independent risk factor for blindness, GHD, impaired thyroid function, and ACTH deficiency (20).

Despite the toxicity in the general population, patient selection may guide the likelihood of severe radiation toxicity. Indeed, in a study of 69 patients with optic chiasm or hypothalamic gliomas that demonstrated excellent 10 year OS and 10 yr PFS 83% and 65.5%, respectively, severe intellectual disability, which occurred in 18 children, correlated strongly with young age of treatment (21).

This retrospective and population-based data is also supported by prospective studies in pediatric LGG including an evaluation of 58 patients with diencephalic tumors that were largely grade 1 optic pathway glioma or pilocytic astrocytoma. Disease control was very good with 5- and 10- year PFS 87% and 74%, respectively, and 5- and 10-yr OS 99% and 96%, respectively. However, these patients had significant late effects of treatment including a decline in cognitive function, with an average of a 10 point decline in intelligence quotient over a five year period for patients age 5 years at treatment. The deleterious effect of radiation diminished with age and ultimately had no impact for patients who were greater than age 12 at time of radiation. Hearing loss was observed in 12%, thyroid hormone deficiency in 64%, and GH deficiency in 49% at 10-yr. Vasculopathy developed in 4.8% of patients at 7 years, with increased risk for younger patients up to 12.5% for the youngest cohort (22, 23).

Further retrospective analysis from other major treatment centers support the conclusion that radiation increases rates of treatment related toxicity and are summarized in **Table 1**. Overall, radiation for diencephalic glioma in the era of photon radiation is associated with very good 15 year overall survival (82%-100%) and local control (40%-63%). However, this often comes at the cost of high incidence of cognitive dysfunction (26-30%), endocrine disruption (26-85%), obesity (45-75%), thyroid hormone deficiency (48%-68%), GH deficiency (39%-100%), and ACTH deficiency (55.6%). Long term risks also include the less frequent but debilitating complications of secondary tumor formation (9-22%), vasculopathy (1-13%), and blindness (27%-57%) (18–23, 25–28). Unfortunately, these long term risks prove fatal in a significant portion (~14%) of patients (10).

**Table 1:** Toxicity associated with photon and proton therapy for Optic Pathway/Hypothalamic Glioma.

Study	Modality	Patients (n)	Median follow up (years)	5y OS/LC	Hearing loss	Endocrine deficiency	Growth HormoneDeficiency	Secondary tumor	Vasculopathy	Radionecrosis
Williams, 2018 (10)	Photons	29	17.8	89%/82%	1%	26%		22%	13%	4%
Armstrong, 2011 (20)	Photons	361	15		3.3%***		60%			
Fouladi, 2003 (24)	Photons	73	6.3	69%/68%**		85%	60%			
Merchant, 2009 (22)	Photons	78	7.4		12%		46% 5-yr, 49% 10-yr			
Tsang, 2017 (25)	Photons*	89	12.5					9%		
Tao, 1997 (26)	Photons	29	6.2	100%/95%		72%	59%			
Brauner, 1990 (27)	Photons	21				100%	100%			
Collet-Solberg, 1997 (28)	Photons	38	5.6				39%			
Indelicato, 2019 (12)	Protons	174	4.4	93%/89%	2%	22%	18%		1%	1%
Greenberger, 1997 (29)	Protons	32	7.6	100%/90%		~75%			6%	
Hug, 2014 (30)	Protons	15	3.3	93%/87%		27%				

Toxicity data is included as described in available publications. Missing datapoints are represented by empty cells. *These patients were treated with 95% photon and 5% proton plans due to logistical constraints. **Data is reported as 6-year OS and LC. ***The rate of hearing loss is increased to 20.3% among patients with tumor progression.

### Proton therapy for optic pathway/hypothalamic glioma

The significant toxicity of photon radiation has led to substantial interest in limiting normal tissue dose using proton therapy. As a first step toward evaluating the benefit of protons in this disease, dose distribution studies comparing proton, 3D photon and lateral photon plans for patients who received proton irradiation for OPGs demonstrate that protons significantly improve the conformity index (CI) and reduce radiation dose to normal tissue compared to either photon technique, with the most significant reductions in dose to the contralateral optic nerve (47% reduction compared to the 3D photon plan and 77% reduction compared to the lateral photon plan), and less dramatic but still consequential reductions to the optic chiasm, with 11% and 16% reductions compared to the 3D and lateral photon plans, respectively, and the pituitary, with 13% and 16% reductions compared to the 3D and lateral photon plans, respectively. In this analysis, larger tumors correlated with increased benefit from proton planning (31).

Clinical studies also support these benefits of proton radiation. The University of Florida experience includes 174 pediatric patients with nonmetastatic LGG treated with proton beam radiation. 52% of these patients had diencephalic tumors. At median follow up 4.4 years, this group had very good treatment outcomes, with 93% 5y OS, 88% 5y PFS, and 89% 5y LC, which compares favorably with the same institution’s data with photons described above (89% 5y OS, 82% 5y LC). This treatment also had a favorable toxicity profile, as hormone deficiency developed in only 22% of patients (compared to 26% treated with photons), and sensorineural hearing loss in 4 patients, with 1 requiring hearing aid. Serious long term toxicity occurred in 7/174 (4%) of patients, including one secondary malignancy, one optic retinopathy, three vasculopathies, and two episodes of brainstem necrosis. However, this too compares favorably with the rate of treatment related death demonstrated in patients treated with photons at the same institution (14%) (12).

A second retrospective review of 32 patients with LGG treated with proton radiation with a longer median follow up 7.6 years including 18 patients with supratentorial tumors (56%) also found favorable outcomes. In this series, 59.4% of patients had pilocytic astrocytoma and only 18.8% had WHO Grade 2 tumors. Of note, 28% of these patients were treated with 80% photons due to scheduling difficulty with the cyclotron. Nevertheless, 6-year and 8-year PFS were consistent with that observed for photon irradiation at 89.7% and 82.8%, and 8 year OS was impeccable at 100%. The authors note a decline in neurocognitive function among patients 4.8-5.4 years of age, although three of these four patients were also in the “high risk” group that received over 15 Gy to 20% of the volume of the left temporal lobe or hippocampus. In addition, two patients with NF1 developed moyamoya (6%). Among patients with supratentorial tumors, greater than 70% experienced endocrine deficiency. Visual symptoms developed in a few patients, and they improved in seven others due to tumor regression (29). A retrospective review of 15 pediatric patients with diencephalic LGG treated with protons at Loma Linda found comparable results, with 3.3yr local control (LC) of 87% and 3.3yr OS 93%. In this cohort, 4/15 developed hypopituitarism requiring hormone replacement (30).

Overall, these results compare favorably with the data from photon therapy, with each study of proton therapy demonstrating excellent tumor control and overall survival. Conclusions regarding long term toxicity will require additional time, as the median follow up for the proton therapy studies is currently insufficient for comparison to the photon therapy studies. However, the early data on adverse events is promising and certainly warrants further investigation (10).

## Craniopharyngioma

Although craniopharyngioma is a benign tumor, it is clinically quite challenging due to the proximity of the optic chiasm and hypothalamus. This tumor was historically treated with surgery alone, which carries risks of damage to these critical structures as well as a risk of recurrence requiring radiation (2, 32–35). STR with adjuvant radiation is an alternative treatment approach that provides substantial disease control and may reduce acute treatment morbidity (2, 3). These tumors may also be treated with radiation alone in patients whose tumors are not amenable to surgery (36). These approaches are highlighted in a large meta-analysis including 531 patients with craniopharyngioma treated with GTR or STR with adjuvant radiation which supports the utility of all of these treatment options in demonstrating equivalent 5-yr PFS (77% vs 73%, respectively) for GTR and STR with radiation and a significant benefit to the addition of radiation in patients who received STR (73% vs 43%) (37).

### Photon therapy for craniopharyngioma

Although GTR is associated with the highest rates of new endocrine dysfunction, panhypopituitarism, and new neurologic deficits (38), multiple high quality retrospective reviews demonstrate that EBRT with photons is also associated with significant morbidity (2) including loss of visual acuity (0%-53%), endocrinopathy (46%-100%), panyhypopituitarism (33%-55%), radionecrosis (7%), and secondary malignancy (4-7%) (**Table 2**) (2, 38–43, 47). Unfortunately, the rate of severe vasculopathy is particularly increased for craniopharyngioma compared to other intracranial tumors, with rates of 5-32% for patients with craniopharyngioma compared to 2-4.3% for patients without suprasellar tumors (2, 39, 41–43, 47, 48). This increased risk corresponds with the close proximity of craniopharyngiomas to the optic chiasm and Circle of Willis and is consistent with the results of much broader studies evaluating the impact of intracranial radiation on stroke incidence. In one such study of 2,202 5-year survivors of childhood cancer with a median follow up of 26 years, El-Fayech, et al. found that pediatric radiation overall is associated with an 8.5-fold increase in risk of stroke. This risk is increased to 15.7-fold for patients with a dose of 40Gy to the Circle of Willis, which is in such close proximity to the optic chiasm that the dose to the chiasm is often used to approximate the dose to the Circle of Willis. In addition, radiation of 10Gy to the Circle of Willis was found to lead to an 11.3% incidence of stroke before the age of 45 (49). It is therefore critical to provide anticipatory guidance and close vascular follow up for patients who receive substantial radiation (e.g. >50 Gy) to the Circle of Willis.

**Table 2:** Toxicity associated with photon and proton therapy for Craniopharyngioma.

Identifier	Modality	Patients	Median follow up (y)	Timeframe	3y OS	5y LC	Endocrine deficiency	Secondary tumor	Vasculopathy	Loss of visual acuity	Obesity	DI
Merchant, 2002 (2)	GTR or STR	15	6	1984-2001		40%	100%			47%		73%
	STR/Bx + Photons	15	6			100%	93%			33%		33%
Fouda, 2020 (39)	STR + Photons	178	10	1960-2017				4%	11%			
Ravindra, 2021 (40)	GTR	31	5.83	1997-2018	100%	73%	96%	2.5%*		20%	70%	90%
	STR/CD + Photons	14			100%	54%	75%			0%	71%	61%
	STR + Protons	11			100%	100%	100%	2.5%	9%	42%	71%	85%
	CD + Protons	7			100%	**77%**	50%		14%	10%	63%	50%
Winkfield, 2011 (41)	STR+Photons	43	8.6	1976-2003		88%	67%	7%	9%	53%	56%	43%
Lo, 2016 (42)	MSR + Photons	19	19	1971-2010			100%		32%			70%
Bishop, 2014 (43)	CD/STR + Protons	21	5	2007-2012	94%		76%		10%	5%	19%	
	CD/STR + Photons	31		1996-2007	97%		77%		10%	13%	29%	
Jimenez, 2021 (44)	STR + Protons	77	4.8	2002-2018		90%	94%		6%			36%
Indelicato, 2017 (45)	STR + Protons	45	2.6	2008-2016	100%				4%			
Hall, 2018 (46)	STR + Protons	135	3	2006-2015					19%			

GTR, gross total resection; STR, subtotal resection; CD, cyst decompression; MSR, maximal safe resection. *One patient in the GTR group developed a secondary malignancy five years after receiving salvage proton therapy for recurrent disease.

### Proton therapy for pediatric craniopharyngioma

Dosimetric comparison of VMAT and pencil beam scanning plans for patients with craniopharyngioma show that pencil beam scanning significantly reduces dose to brain substructures associated with cognition in and outside of the temporal lobe including the hippocampus (50). In addition, comparison of intensity modulated radiation therapy (IMRT), 3D-proton radiation therapy (PRT), and intensity-modulated proton therapy (IMPT) plans demonstrates that both types of proton plans result in significant reduction of dose to hippocampus, dentate gyrus, subventricular zone, nearby vasculature, and uninvolved brain outside the planning target volume (PTV) (51). In comparisons of proton IMPT, double proton scatter (DPS), and photon IMRT plans throughout treatment, IMPT demonstrates significantly improved conformity index (CI) and significantly reduced normal tissue dose compared to both DPS and IMRT. When comparing IMRT and DPS, IMRT had higher CI and lower optic nerve dose, but DPS exhibited lower doses to the optic chiasm, normal brain, and cochlea with a reduction in mean planning target volume of 11.3%. Taken together, this evidence suggests that while any tested method of proton therapy reduces doses to critical brain structures compared to IMRT, IMPT results in the most substantial dose reduction (52).

Multiple retrospective reviews also support the use of proton therapy in patients with craniopharyngioma. One such comparison included 63 pediatric patients with craniopharyngioma treated between 1997 and 2018 with either GTR alone, STR or cyst decompression (CD) + IMRT, STR + proton beam therapy (PBT), or CD + PBT. Of note, the IMRT data in this study was not stratified by extent of resection because 12/14 patients in the IMRT group received CD + IMRT, making the data in this group most consistent with CD + IMRT. At five years follow up, PFS was statistically and clinically significantly improved in the STR+PBT and CD+PBT groups compared to the CD/STR + IMRT group, with 5-yr PFS 73%, 54%, 100%, and 77% for GTR alone, STR or CD + IMRT, STR + PBT, and CD + PBT, respectively. 5 yr OS was 100% for all groups, but multiple deaths in GTR and IMRT groups occurred before 12 years compared to 100% OS at 17 years among all patients treated with protons. Hypopituitarism was significantly more common the GTR and STR + PBT groups compared to the CD/STR + IMRT and CD + PBT groups at 96%, 100%, 75%, and 50%, respectively, which corresponds with the increased risk of hypopituitarism with increased extent of surgery. Diabetes Insipidus (DI) was similarly elevated in these groups at 90%, 85%, 61%, and 20%, respectively. Although survival outcomes were very good, two patients in the PBT group experienced treatment-related vasculopathy and one developed a secondary malignancy. In addition, one patient in the GTR group developed a secondary malignant neoplasm in the brainstem five years after salvage treatment with proton radiation for recurrent disease. PBT also had non-significantly increased rates of vision loss which developed in 42% of patients treated with PBT compared to only 20% in the GTR group. Although obesity was common after treatment, there were no differences between groups (40).

This result is corroborated with a second retrospective review of 52 patients with craniopharyngioma treated with PBT (21) or IMRT (31) which found similar OS across treatment groups (96% 3yr OS) but no difference in OS, PFS, rate of cyst growth, or toxicity at 59.4 months between the two radiation techniques, with decreased visual acuity in 5% and 13%, vasculopathy in 10% and 10%, and endocrine dysfunction in 76% and 77% of patients treated with protons and photons, respectively (43).

Another study of 77 patients with pediatric craniopharyngioma treated with protons demonstrated similar findings and toxicity. In this group, 18% underwent GTR, 60% STR, and 22% biopsy or CD. Grade 3 toxicity was observed in only 4% of patients. At median 4.8 years from treatment, there were only 6 local failures (6.6%) and 3 deaths (3.3%). Five year local control was 90% among evaluable patients. Proton therapy was associated with infrequent incidence of endocrine deficiency, visual impairments, and moyamoya syndrome which were present after treatment in 94%, 40%, and 11% of patients compared to 87%, 52%, and 6% before treatment. Fortunately, neurocognition was not clinically impacted by proton beam radiation (44).

Data from the UF proton center is similar, with 3 yr OS and PFS both 100% for 45 pediatric craniopharyngioma patients treated with proton therapy. Only one of these patients experienced vision loss (4%) (45). Although only one patient in this cohort experienced symptomatic vasculopathy (4%), a larger study at the same institution found that proton therapy for craniopharyngiomas resulted in development of new vasculopathy in 19.3% of patients (46). As discussed above, this high rate of vasculopathy is likely due to the close proximity of craniopharyngiomas to the Circle of Willis (46, 48).

Overall, the early data on proton therapy for craniopharyngiomas is very promising, with exceptional rates of disease control (100% 5yr OS, 90-100% 5y LC) and infrequent secondary tumor formation within the reported follow up period. Although these data compare favorably to the experience with photon radiation, further research is needed to evaluate the long term risk of vasculopathy and secondary tumor formation in this population (40, 43–45, 52).

## Intracranial germ cell tumors

Intracranial germ cell tumors are a heterogeneous group of tumors that arise from primordial germ cells. Germ cell tumors are divided into germinoma and non-germinomatous tumors, of which germinomas are associated with much better outcomes. Although germ cell tumors most commonly arise in the pineal gland, 20-25% arise in the suprasellar region, and 5-15% are bifocal with simultaneous presentation in the pineal gland and suprasellar region (53–56). In addition to imaging, suprasellar involvement may also be defined by a history of DI. Localized intracranial germinoma, including bifocal tumors, can be cured by whole ventricle radiation and local boost radiation without surgery, but the addition of chemotherapy allows de-escalation of radiation doses (57–61). Metastatic germinomas require craniospinal irradiation (CSI) with a local boost (57–60), possibly with chemotherapy. Surgery may be utilized in the setting of recurrent disease, when resection provides significant benefit (62). Nongerminomatous germ cell tumors (NGGCT) are more treatment resistant and require combination therapy with chemotherapy (usually cisplatin) and adjuvant radiation, typically CSI (63) but with recent consideration of reduced volumes (64), to achieve 5 year event free survival (EFS) and OS of 70-80% (4, 5, 62, 64, 65). As this review aims to understand the toxicities associated with radiation to diencephalic tumors, the following sections focus specifically on the relevant toxicity for those germ cell tumors with suprasellar involvement.

### Photon-based radiation for intracranial germ cell tumors

As seen in low grade glioma and craniopharyngioma, the high cure rates associated with photon-based radiation for germ cell tumors are not without consequence. Among patients treated with photon-based radiation, endocrine disruption is common (33-56%), with incidence increasing to 73% in patients who receive greater than 45Gy to the tumor bed (56, 66, 67). Neurocognitive impairment is also common, with an incidence of 50% in patients with suprasellar disease (56). Secondary malignant neoplasms develop in about 6% of patients with germinoma and 4% of patients with NGGCT (56, 66, 67). In addition, evaluation of data from the SEER database suggests that these patients have a substantially elevated risk of death from cerebrovascular events in the decades following radiation, reinforcing again the importance of close vascular follow up for patients receiving radiation for diencephalic tumors (67).

### Proton-based radiation for intracranial germ cell tumors

Dosimetric analyses of radiation planning for germ cell tumors including focal and whole ventricle radiation have demonstrated significant reduction of dose to normal brain, hippocampus, cerebellum, supratentorial brain, temporal lobes, and frontal lobes with protons (68, 69). Clinical data also support a role for proton therapy in reducing toxicity, although the data in this setting is much more limited.

In one retrospective review of 14 patients with nonmetastatic intracranial nongerminomatous germ cell tumors treated with adjuvant proton therapy after chemotherapy, 8 received CSI, 2 whole ventricle radiation, and 4 focal radiation alone. All patients were alive at 2.8 year median follow up with three year PFS 86% that compares favorably with historical PFS 70-80% with chemoradiation (4). Of the two patients who progressed with metastatic recurrence, both received focal radiation alone. Serious adverse events were limited to the patients who received CSI, among whom 3 experienced loss of visual acuity and one developed growth hormone deficiency (11).

Although this retrospective review did not include rigorous neurocognitive testing, a prospective evaluation of 34 patients with intracranial germ cell tumors found that these patients overall had significant decreases in neurocognitive function compared to the general population at diagnosis but patients with diencephalic tumors had no further deterioration of neurocognitive function after proton therapy (70).

Another retrospective study of 127 patients with CNS germ cell tumors treated with upfront chemotherapy followed by patient’s choice proton or photon radiation shows equivalent survival between the two radiation techniques. Secondary malignancy developed in 6/127 of the patients in this study, resulting in fatality in one patient, but the authors did not differentiate whether the type of radiation impacts this prevalence (71).

Together, this evidence suggests that proton therapy achieves similar rates of tumor control to photon based radiation. However, conclusions on the benefit of proton therapy in this disease site will require additional evidence comparing outcomes for patients treated with photon or proton therapy.

## Pituitary adenomas

Pituitary adenomas are a relatively rare but largely benign tumor in the CNS. About 2/3 of pituitary adenomas are functional. These tumors most commonly secrete prolactin leading to galactorrhea, amenorrhea, and menopausal symptoms, but can also secrete other hormones include growth hormone (GH) leading to acromegaly or ACTH leading to Cushing disease. It is also possible for these tumors to secrete TSH, although this is a much rarer phenomenon.

### Gamma knife for pituitary adenomas

Although functional pituitary adenomas are often cured with surgical resection, radiation is required for salvage therapy in about 20-40% of cases (7, 8). The treatment of choice in these cases has historically been gamma knife (GK) radiosurgery, which allows precise targeting of high doses of radiation in 1-3 treatments.

This technique has produced very good outcomes, as exemplified in a review of 418 patients treated with GK demonstrating a tumor control rate of 90%, with median follow up of 31 months and median time to endocrine remission of 48.9 months. However, this therapy is not without complication as it produces new onset pituitary hormone deficiency in 8-24% of treated patients, permanent cranial nerve deficit in 0.5% of treated patients, and loss of visual acuity in 2% (13, 72).

### Proton therapy for pituitary adenomas

A subset of patients with pituitary adenoma may not be ideal for treatment with GK due to the proximity of the optic chiasm. Such patients may be more appropriately treated with fractionated stereotactic radiosurgery (SRS). In a dosimetric comparison of treatment plans, stereotactic proton therapy reduced dose to optic nerve, hypothalamus and normal brain compared to stereotactic photon therapy. Given our current understanding of the risks of secondary malignancy, this difference corresponds with a reduction of projected risk of secondary malignancy from 12.93% to 5.28% (p=0.008) (73).

Proton based SRS has also been utilized in the clinic, including in a study of 165 patients with functional pituitary adenomas refractory to resection (162 patients) and/or photon radiation (3 patients) who were treated with proton stereotactic radiosurgery (92% of patients) or fractionated proton therapy (8%). In these patients, tumor control was 98% at 43 months, which compares favorably with the 90% control rate expected for GK SRS. In addition, biochemical complete responses were achieved by 3 years in 54% of patients with Cushing disease with a time to endocrine remission of 32 months, 63% of patients with Nelson syndrome with a time to endocrine remission of 27 months, 26% of patients with acromegaly with a time to endocrine remission of 62 months, and 22% of patients with prolactinomas with time to endocrine remission of 60 months. Actuarial 3 and 5 year rates of new hypopituitarism were 45% and 62%, with larger radiated volume correlating with higher risk. Four patients had new onset seizure after radiation. Of note, treatment in this study included the entire sella turcica, but attempts are now being made to reduce toxicity further by narrowing the treatment field (74).

## Discussion

The combination of stark differences in dose distribution and increasingly impressive clinical data has resulted in a consensus among practitioners that proton based radiation therapy is the preferred choice for children with diencephalic tumors. This conclusion is documented in the Consensus Report from the 2015 Stockholm Pediatric Proton Therapy Conference, which reports universal agreement that protons should be used in patients with craniopharyngioma, low grade glioma, and optic pathway glioma. In contrast, photons were preferred in the case of high grade glioma, in which very poor outcomes negate the long term benefit of reducing toxicities. As part of the conference, photon plans and proton plans were created by experienced radiation oncologists at centers that predominantly complete photons and proton plans, respectively. Even in this setting, proton plans produced significantly reduced normal tissue doses including significant reduction of dose to supra-tentorial brain in patients with craniopharyngioma, reflecting the benefit of proton planning in these patients (75).

Future work in this area should include further improvement of proton based radiation techniques to improve conformality and reduce normal tissue toxicity. These efforts will be aided by additional research that identifies the most critical structures to avoid and evaluates the impact of proton therapy on long term outcomes. For example, retrospective analyses have identified significant positive correlations of vascular toxicity with dose to the optic chiasm, suggesting that planning that minimizes dose distribution to critical vasculature may further reduce this complication. Linear energy transfer (LET) based methods of treatment planning may provide new strategies for further reducing biologic dose to this critical vasculature (46, 76).

Several technologic developments offer the potential to further reduce late effects from proton therapy. IMPT is increasingly clinically available and offers improved conformality over passive scatter proton therapy. Stereotactic proton therapy is emerging as a treatment technique that may offer benefits for selected pediatric patients. On the horizon, FLASH proton therapy enables the delivery of very high doses of radiation in fractions of a second. Early clinical results suggest that this treatment can be delivered safely and can reduce radiation-mediated damage to normal tissues (77–79). Further basic, translational, and clinical research investigations into the potential of FLASH therapy to reduce late effects, especially in children, are eagerly awaited.

One of the challenges in radiation treatment recommendations is the late onset of severe radiation toxicity, which occurs most frequently and sometime exclusively many years after treatment. It is likely that treatment recommendations will be made before the full toxicity of novel treatments is known. In those cases, it is essential to continue rigorous analysis of retrospective data to determine impacts on patient outcomes.

## Conclusion

Radiation is often an essential modality for long-term control of pediatric diencephalic tumors but is challenging due to the close proximity of critical adjacent structures including vasculature, pituitary gland, optic chiasm, and optic tracts. Proton therapy can mitigate these concerns by enabling more precise delivery of radiation to tumor targets while minimizing dose to normal brain tissue. More time will be needed to determine the long term outcome of patients treated with proton therapy, but early clinical data suggest that proton therapy is safe and effective for pediatric diencephalic tumors. Future work will include further advances in radiation technology including IMPT, proton-based SRS, and FLASH-proton therapy, which each promise to decrease normal tissue toxicity without compromising tumor control. Coordination between major treatment centers will likely be necessary to evaluate each of these approaches for safety and efficacy as they become more widely available.

## Author contributions

AG and SM designed the study, wrote, and edited the manuscript. All authors contributed to the article and approved the submitted version.
